# CysLT1 receptor antagonist alleviates pathogenesis of collagen-induced arthritis mouse model

**DOI:** 10.18632/oncotarget.22664

**Published:** 2017-11-26

**Authors:** Minwen Xu, Ruiyun Hong, Xiaoli Zhang, Hailin Zou, Yi Zhang, Zhiping Hou, Liefeng Wang

**Affiliations:** ^1^ First Affiliated Hospital, Gannan Medical University, Ganzhou, China; ^2^ Department of Biotechnology, Gannan Medical University, Ganzhou, China

**Keywords:** collagen-induced arthritis, cysteinyl leukotrienes, CysLT1 antagonist, montelukast, IL-17A, Immunology and Microbiology Section, Immune response, Immunity

## Abstract

Cysteinyl leukotrienes (CysLTs) play a key role in inflammatory diseases such as asthma and their receptors’ antagonists are currently used as anti-asthmatic drugs. CysLTs have also been found to participate in other inflammatory reactions. Here, we reported that in rheumatoid arthritis (RA) animals model, collagen-induced arthritis, (CIA), CysLT1, a receptor for CysLTs, was up-regulated in hind paw and lymph node, while CysLTs levels in the blood were also higher than normal mice. Montelukast, a drug targeting CysLT1, has been shown to effectively reduce the CIA incidence, peak severity, and cumulative disease scores. Further study indicated that CysLT1 signaling did not affect the differentiation of pathogenic T helper cells. We conclude that montelukast may play important roles in the pathogenesis of CIA, mainly by inducing infiltration of pathogenic T cells, increasing IL-17A secretion and expression of IL-17A, while these effects can be blocked by CysLT1 antagonists. Our findings indicate that antagonist of CysLT1 receptor may be used to treat rheumatoid arthritis.

## INTRODUCTION

Rheumatoid arthritis (RA) is a chronic inflammatory disease characterized by immune-mediated synovitis, local inflammatory cells infiltration and neoangiogenesis [1, 2]. Although the etiology of the disease remains unclear, it is considered a complex chronic disorder which inflammatory mediators played a crucial role in the rheumatoid synovium and synovial fluid [3]. IL-17A, an important proinflammatory cytokine, has been reported to participate in RA pathogenesis [4]. IL-17 receptors are positive in progenitor cells from RA cartilage, IL-17A/F increase the RUNX2 and IL-6 protein expression levels and up-regulate the MMP3 mRNA expression levels. Anti IL-17 antibody reduced the secretion of IL-6 protein level and increased the secretion of IL-10 protein level [5]. Knocking out IL-17A or treatment with anti IL-17 antibodies or blocking IL-17 receptor may alleviate arthritis [6-8]. Of course more clinical trials are need to confirm those effect since occasionally contradictory results with IL-17 were found in certain RA models [9].

G protein-coupled receptors (GPCRs) mediate many important biological pathways and are targeted by the largest proportion in the current medicine market [10, 11]. Many GPCRs have been shown to link to the pathogenesis of RA [12-14].

Leukotrienes (LTs), an endogenous signaling molecule, are potent pro-inflammatory mediators which involve in host defense and some inflammatory diseases [15-18] via designated GPCRs. Arachidonic acid is sequential catalyzed by cytosolic phospholipase A2α (cPLA2α), 5-lipoxygenase (5-LO) and LTC4 synthase (LTC4S). LTC4, LTD4 and LTE4 are released successively which are called CysLTs [19, 20]. The physiological functions of CysLTs are mediated via two CysLTs receptors, CysLT1 and CysLT2 [21, 22]. Inhibitors of 5-LO and antagonists of CysLT1 are used in treatment of asthma [23, 24].

The role of CysLT1 signaling pathway in the pathogenesis of RA is unclear. In our study, we discovered in CIA mouse, a RA animal model, *cPLA2α*, *5-LO* and *CysLT1* mRNA levels were up-regulated, and CysLTs levels in serum were also elevated. Blocking of CysLT1 with its antagonist, montelukast, relieved the CIA’s clinical symptoms. We demonstrated that CysLTs signaling pathway play a vital role in the pathogenesis of CIA, primarily by up-regulating the expression of *IL-17A* gene, then inducing production of IL-17A, and infiltration of pathogenic T cells which can be relieved by blocked CysLT1 signaling pathway. Our results demonstrate that the CysLT1 antagonists may be used to treat RA.

## RESULTS

### Key ingredients of CysLT1 signaling pathways were up-regulated in the pathogenesis of CIA

**Figure 1:**
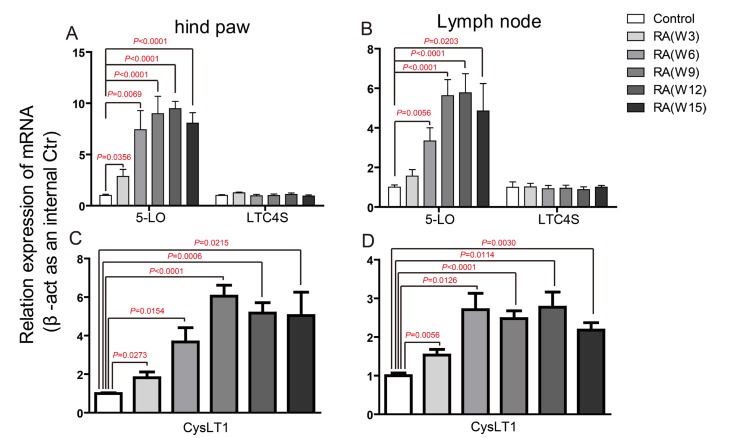
Up-regulation of CysLT receptors and synthesizing enzymes during CIA pathogenesis mRNA was isolated from hind paw, lymph node of control mice and CIA mice at weeks 3, 6, 9, 12, 15 post booster immunization. qPCR was performed to analyze gene expression. Results were normalized to β-actin expression in the same sample and then normalized to the control. **A.**-**D.** *5-LO* and *LTC4S* gene expression in hind paw (A), lymph node (B), *CysLT1* gene expression in hind paw (C), lymph node (D). Data are presented as mean ± SEM (n=6) and are representative of three independent experiments. *p < 0.05, **p < 0.01 and ***p < 0.001, versus control (Student’s *t*-test).

CIA was induced in DBA male mice by immunization with Chicken type II collagen [25]. To describe the role CysLTs played in the pathogenesis of CIA, we firstly detected the mRNA levels of key ingredients in CysLT1 signaling pathways, including the receptor CysLT1 and three CysLTs synthesizing enzymes (cPLA2α, 5-LO and LTC4S) in hind paw and lymph node at week 0, 3, 6, 9, 12, 15 post booster immunization (Figure 1 and Supplementary Figure 1). In hind paw, the *cPLA2α* mRNA was increased at week 3 and week 6 post booster immunization (Supplementary Figure 1A), the *5-LO* mRNA level was observably increased from week 3 post booster immunization till reached its plateau at week 6 (Figure 1A). In lymph node, the *cPLA2α* mRNA was increased at week 3 and week 6 post booster immunization (Supplementary Figure 1B), the level of *5-LO* mRNA increased from week 6 and maintained a relatively stable level after week 9 (Figure 1B). In contrast, *LTC4S* were not significantly altered in both hind paw and lymph node.

**Figure 2:**
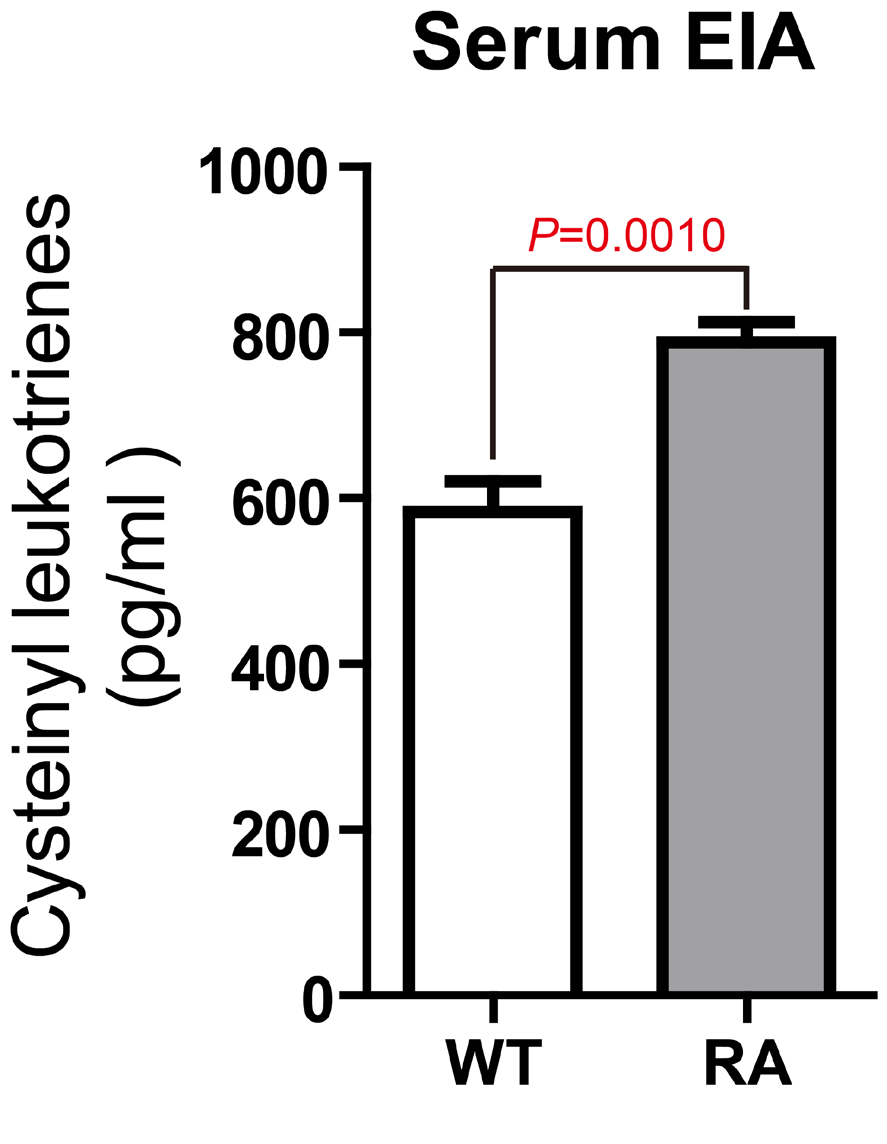
Significantly increased CysLTs levels in the serum of CIA mice Total CysLTs concentrations in serum (6 weeks post booster immunization) were measured with an EIA kit. Data are presented as mean ± SEM (n=3) and are representative of three independent experiments. *p < 0.05, **p < 0.01, versus control (Student’s *t*-test).

*CysLT1* was found to be significantly up-regulated from week 3. In hind paw, *CysLT1* peaked at week 9, and then slowly declined after that (Figure 1C). In lymph node, however, CysLT1 peaked at week 6 and displayed a plateaued up-regulation between week 6 and week 15 (Figure 1D). We then measured the level of total CysLTs (LTC_4_, D_4_, E_4_) in CIA mice. Compare to control mice, the CysLTs levels in serum (Figure 2), in hind paw (Supplementary Figure 2A) and in lymph node (Supplementary Figure 2B) of CIA mice (6 weeks post booster immunization) were significantly increased. The up-regulation of key ingredients of CysLT1 signaling pathways indicates that CysLT1 signaling pathways may play a crucial role in CIA pathogenesis.

### Treated with CysLT1 antagonists alleviated clinical symptoms and lesion with infiltration of pathogenic T cells in CIA

**Figure 3:**
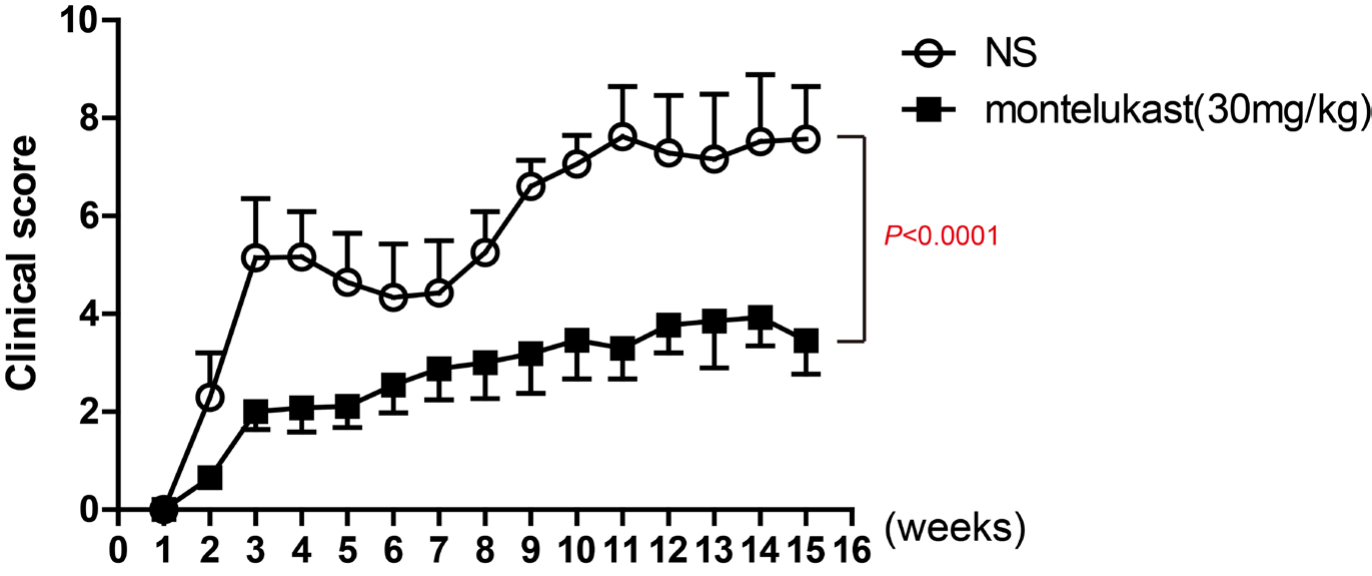
CysLT1 receptor antagonists alleviate pathogenesis of CIA CIA was induced in male DBA mice by immunization with chicken type II collagen. Drugs (montelukast 30mg/kg/d) were given once daily via *i.p.* injection from day 1 post booster immunization till the end of the study and clinical scores were recorded. Control groups were treated with 0.9% saline injections. Data are presented as mean ± SEM (n=6) and are representative of three independent experiments. *p < 0.05, **p < 0.01, versus vehicle control (Mann-Whitney test).

To ulteriorly estimate the participation of CysLT1 signaling pathways in CIA pathogenesis, montelukast, one of CysLT1 receptor antagonists, was used to treat in CIA mice. Montelukast was injected once daily via *i.p.* while vehicle was injected as the control from day 1 post booster immunization till the end of the experiments. The results clearly showed montelukast significantly ameliorated the severity of CIA (Figure 3).

**Figure 4:**
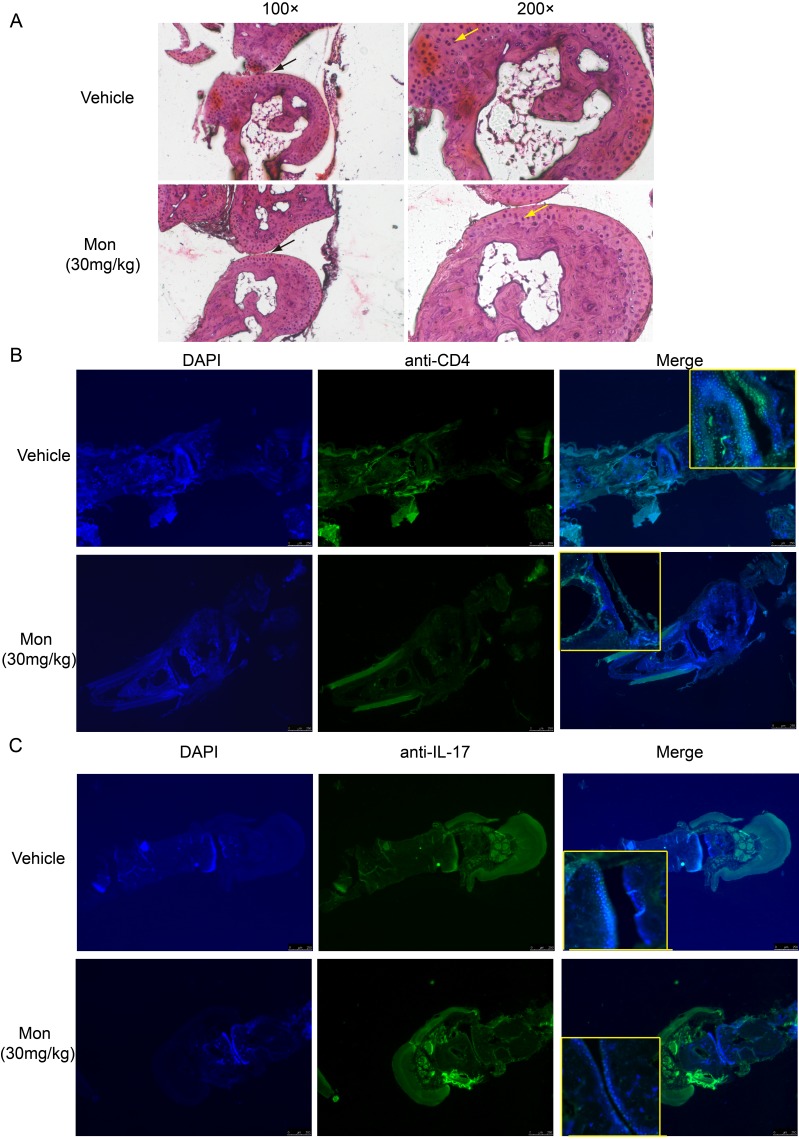
Montelukast treatment reduces pathogenic T cells in the hind paw H&E staining of paraffin sections of hind paw isolated from vehicle or montelukast (30 mg/kg, starting from day 1 post booster immunization) treated CIA mice. Black arrows indicate the bone changes, yellow arrows indicate the areas of Infiltration **A.** Immunofluorescent staining of CD4+ T cells **B.** and IL-17A+ T cells **C.** in the paraffin sections of hind paw isolated from vehicle or montelukast (30 mg/kg) treated CIA mice.

Histopathological examination of hind paw was performed at weeks 9 post booster immunization. Compared to vehicle treatment, montelukast decreased synovial hyperplasia, inflammatory cells infiltration into synovium and bone involvement (Figure 4A). The lesion leukocytes infiltration in hind paw sections were further analyzed by immunofluorescent staining and in synovial fluid were analyzed by flow cytometry. Montelukast treatment could reduce the number of CD4^+^ T cells (Figure 4B, Supplementary Figures 3A and 4A) and IL-17A^+^ T cells (Figure 4C, Supplementary Figures 3B and 4B) in the hind paw sections and in synovial fluid, which were consistent with H&E staining result. We also checked the anti-collagen II auto-Abs ELISA levels in two experimental groups. The results showed that montelukast could decrease the levels of anti-collagen II auto-Abs (Supplementary Figure 5).

### CysLT1 signaling did not influence T cell proliferation and differentiation

**Figure 5:**
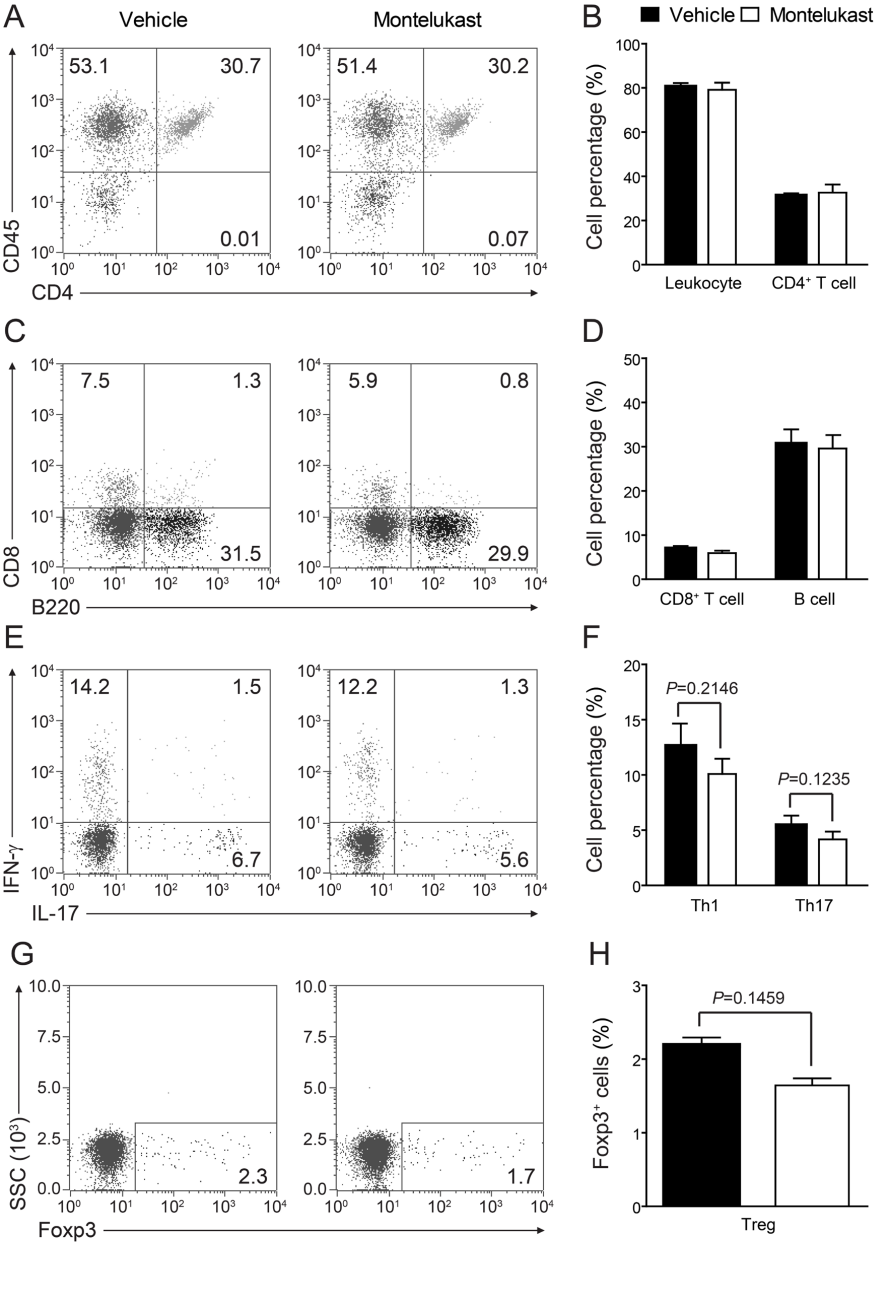
Montelukast treatment does not affect T cell proliferation Leukocytes in peripheral blood were isolated from CIA animals treated with montelukast (30 mg/kg) or vehicle control at weeks 9 post booster immunization and analyzed with flow cytometry. **A.**-**D.** Surface staining of CD45^+^ cells, CD4^+^ T cells, CD8^+^ T cells and B cells; **E.**-**H.** Th1, Th17 and Treg cells were analyzed by intracellular staining of IFN-γ, IL-17 and Foxp-3 respectively within the CD4^+^ gate. Data represents mean ± SEM of three independent experiments.

**Figure 6:**
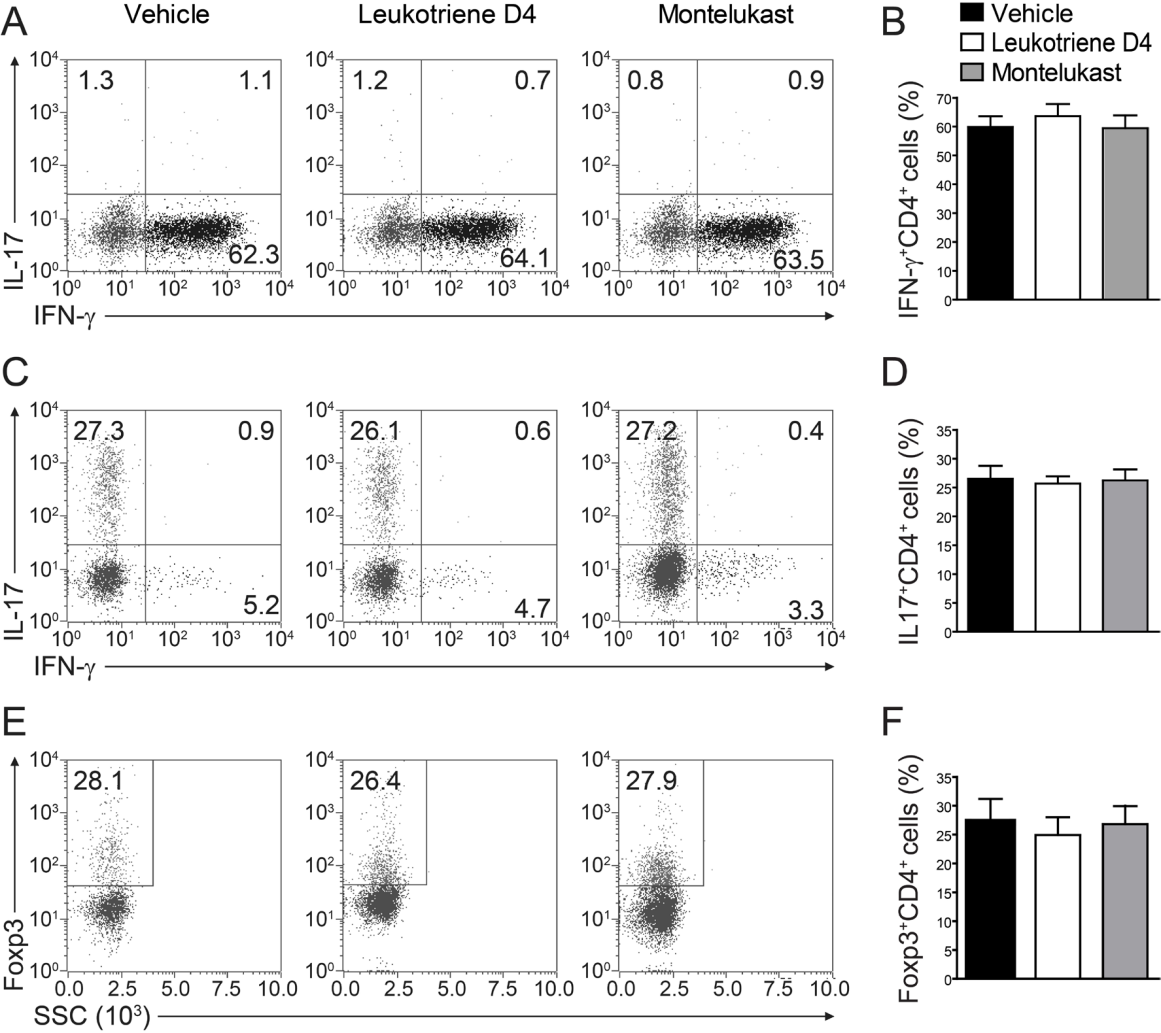
Montelukast treatment does not affect T cell differentiation Naive CD4^+^ T cells isolated from spleen of 8-9 week-old naive mice were induced to differentiate into Th1 **A.**, **B.** Th17 **C.**, **D.,** or Treg **E.**, **F.** *in vitro*, in the presence of leukotriene D_4_ (100μm) or montelukast (100μm). Data represents mean ± SEM of three independent experiments.

We are curious to learn whether CysLT1 signaling pathway was participate in T cell proliferation and differentiation. In CIA animals, montelukast did not obviously decrease the percentage of total leukocytes (CD45^+^ cells), CD4^+^ T cells, CD8^+^ T cells and B cells in blood (Figure 5A-5H). We found there were no differences in the frequency of Th1, Th17 or Treg cells in the CD4^+^ population between two experimental groups, though the trends of decrease could be observed. We also checked T cell proliferation in spleen (Supplementary Figure 6A) and lymph node (popliteal and inguinal, Supplementary Figure 6B) and there were no differences in experimental groups. We further checked the total cells number in spleen and lymph node (Supplementary Figure 7). Cell numbers were no change in spleen (Supplementary Figure 7A) but some change in lymph node (Supplementary Figure 7B). We then checked the absolute cell numbers of B cells, CD4^+^ T cells and CD4^+^ T cells’ subsets in lymph node. We found that CD4^+^ T cells (Supplementary Figure 7D), Th1 and Th17 cells (Supplementary Figure 7E) number were decreased, while B cells (Supplementary Figure 7C) and Treg cells (Supplementary Figure 7E) number were no change. We further labelled T cells with CFSE in lymph node and we confirmed that montelukast did not influence T cell proliferation (Supplementary Figure 8). Since Ki67 is the proliferation related markers, we check the CD4^+^Ki67^+^ T cells and we found that montelukast did not influence the CD4^+^Ki67^+^ T cells number in experimental groups (Supplementary Figure 9). To exclude that montelukast may induce apoptosis, we checked the apoptosis related marker (Bcl-2 and Caspase-3) and we found that montelukast did not expedite apoptosis (Supplementary Figure 10). We further exam whether CysLTs or the antagonist montelukast would affect Th1, Th17 or Treg differentiation *in vitro*. Naive CD4^+^ T cells were activated with anti-CD3 and anti-CD28 antibodies, and then induced to differentiate into Th1, Th17 or Treg cells by supplementation with differentiation factors and LTD_4_ (100μm) or montelukast (100μm). Cells were harvested 3 days later and performed intracellular staining for IFN-γ, IL-17, or Foxp3 [22]. Upon FACS analysis, we found that both LTD_4_ and montelukast did not influence the differentiation of Th1 (Figure 6A, 6B), Th17 (Figure 6C, 6D) or Treg (Figure 6E, 6F) *in vitro*. To exclude the possible that high dose of IL-12 may mask the effect of montelukast treatment, we titrated down the concentration of IL-12 for in *vitro* Th1 differentiation. Different dose of IL-12 were tried and there were no differences for in *vitro* Th1 differentiation (Supplementary Figure 11). Then we titrated down the concentration of montelukast during CD4^+^ T cells differentiation and there was indistinguishableness in different dose of montelukast (Supplementary Figure 12). Recently, T follicular helper (Tfh) cells have been researched as a new T helper lineage and were associated with auto-antibodies in CIA [26]. We checked the Tfh cells (Bcl6^+^CXCR5^+^CD4^+^ T cells) in spleen and lymph nodes after treated with montelukast in CIA mice and we found that montelukast had no any effect in Tfh cells proportion and Tfh absolute cells number (Supplementary Figure 13). The preliminary results may indicate that montelukast had no effect on Tfh cells although more evidences were need.

### Montelukast decreased the secretion of IL-17A and the expression of *IL-17A* gene

**Figure 7:**
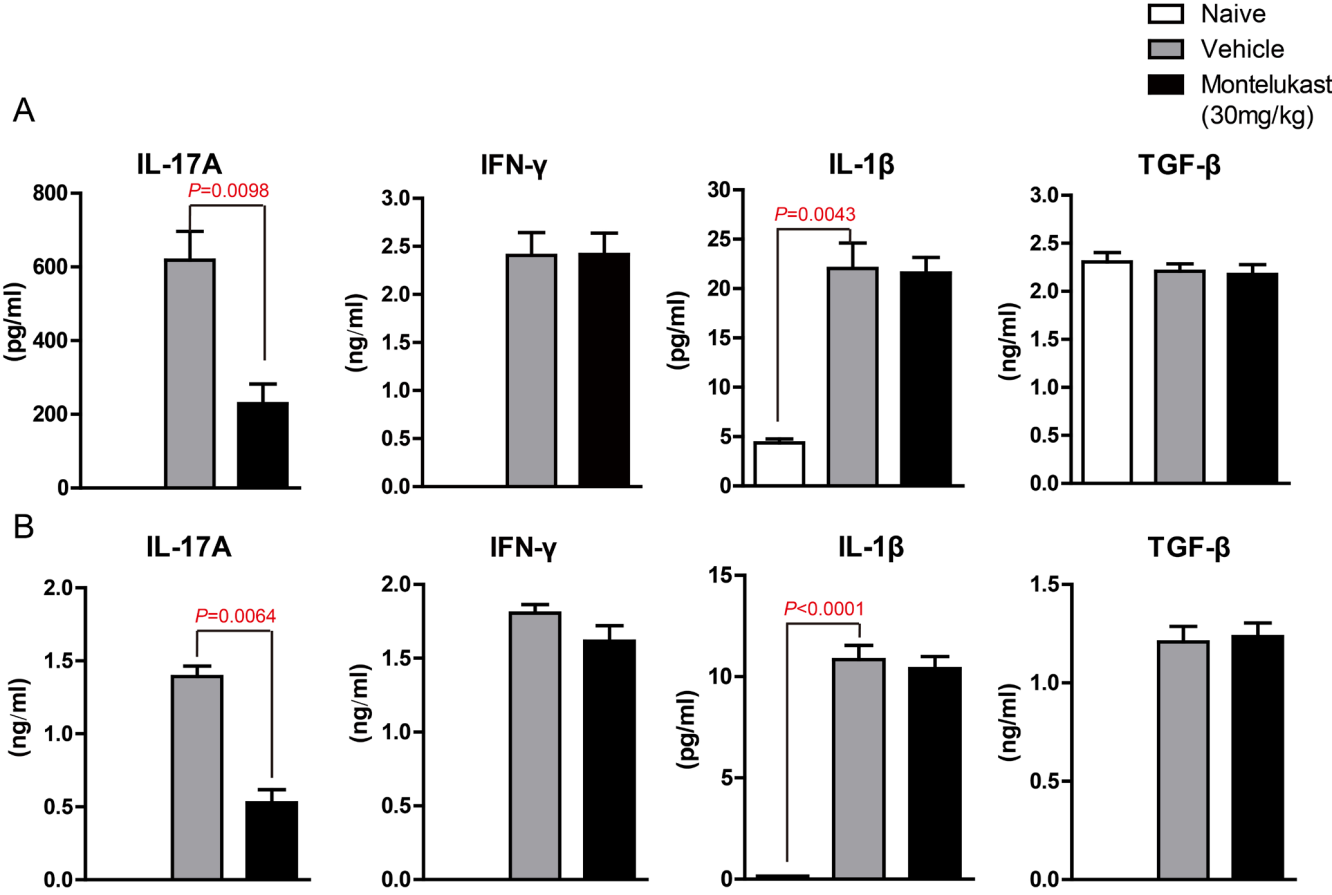
Montelukast decreased the production of IL-17A in CIA mice naive and CIA mice treated with vehicle or Montelukast serum **A.** and homogenate supernatant **B.** were collected and the amounts of IL-17A, IFN-γ, IL-1β and TGF-β were determined using specific ELISA. Data are presented as mean ± SEM (*n* = 3) and are representative of three independent experiments. *p < 0.05, **p < 0.01, versus control (Student’s *t*-test).

**Figure 8:**
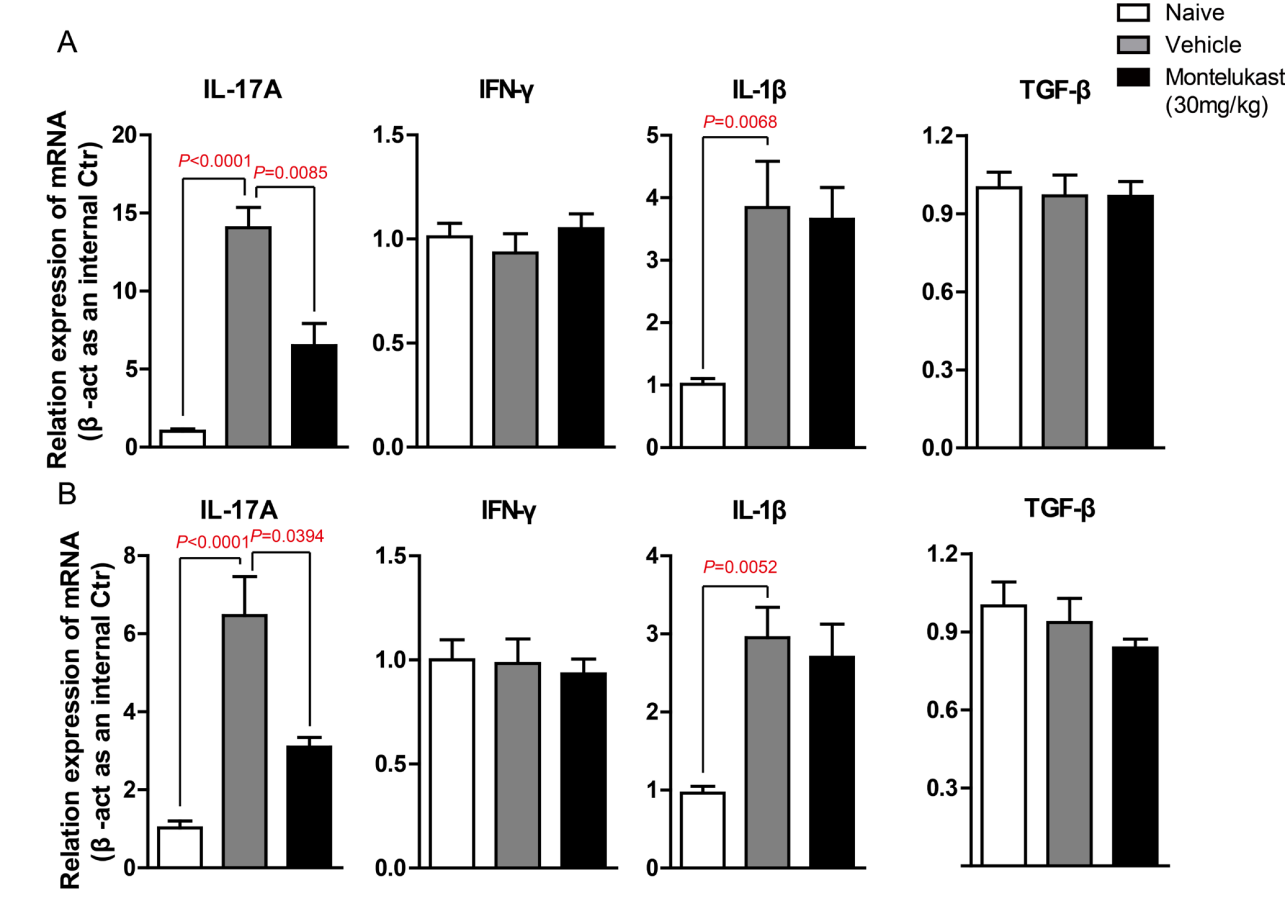
Montelukast down-regulate the expression of IL-17A gene in CIA mice. **A.** Leukocytes in peripheral blood were isolated from naive mouse, CIA animals treated with montelukast (30 mg/kg) or vehicle control at weeks 9 post booster immunization, *IL-17A*, *IFN-γ*, *IL-1β* and *TGF-β* gene are analyzed by qPCR. **B.** Hind paw of naive mouse, CIA animals treated with montelukast (30 mg/kg) or vehicle control at weeks 9 post booster immunization homogenate. RNA was isolated and *IL-17A*, *IFN-γ*, *IL-1β* and *TGF-β* gene are analyzed by qPCR. Data are presented as mean ± SEM (*n* = 3) and are representative of three independent experiments (. *p < 0.05, **p < 0.01, versus control (two-way ANOVA test).

We are curious to learn whether montelukast can decreased the secretion of cytokines. The serum from CIA mice treated with vehicle or Montelukast was collected and the levels of IL-17A, IFN-γ, IL-1β, TNF-α and TGF-β were detected using specific ELISA Kits. As shown in Figure 7A and S14A, montelukast obviously inhibited the secretion of IL-17A. In contrast, the production of IFN-γ, IL-1β, TNF-α and TGF-β were not significantly affected by montelukast. Then we checked the levels of IL-17A, IFN-γ, IL-1β, TNF-α and TGF-β in hind paw. After homogenization, the hind paw supernatant was collected and the cytokines were tested. As shown in Figure 7B and Supplementary Figure 14B, montelukast obviously inhibited the secretion of IL-17A in hind paw. Still, there were no change in other cytokines. We then checked the expression levels of cytokines. The *IL-17A* mRNA of leukocytes in peripheral blood (Figure 8A) and lesions joints (Figure 8B) were significantly decreased at week 9 after treated with montelukast. In contrast, *IFN-γ*, *IL-1β*, *TNF-α* and *TGF-β* gene were not significantly altered in both vehicle and montelukast treated CIA mice (Figure 8A, 8B, Supplementary Figure 15).

Taken together, we can draw a conclude that CysLT1 signaling pathway is not participate in the differentiation or proliferation of inflammatory T cells or regulatory T cells, but might affect the cytokine secretion, gene expression in Th17 cells and infiltration of pathogenic T cells.

## DISCUSSION

As a systemic autoimmune disease, RA is characterized by immune-mediated synovitis, local inflammatory cells infiltration and neoangiogenesis. Due to insufficient understanding of the pathogenesis of RA, there are still many obstacles in the treatment of RA, and new therapeutic targets need to be determined [27]. As one of the biggest receptor super-family, GPCRs widely take part in all kinds of biological activities and participate deeply in several kinds of diseases [28]. Because of its significance biological function and cell surface localization, GPCRs are the most targeted macromoleculars on the clinical medicine [29]. Recently, SCH-23390, a D1-like receptor antagonist, has been demonstrated to inhibit cartilage destruction [30]. This study showed that dopamine level was obviously increased in RA synovial fluid, dopamine could increase IL-17 secretion via D1-like receptors which was IL-6 depended. In a RA mouse model, SCH-23390 intensively decreased the aggregation of IL-6^+^ and IL-17^+^ T cells with alleviated cartilage destruction. This study assesses the role of a D1-like receptors in the pathogenesis of Th17-mediated autoimmune disease, RA. Another report showed that dopamine D2 receptor was expressed on B cells in RA synovial tissue, which has been found to be negatively correlated with the levels of TNF-α and with clinical manifestation [31]. Other GPCRs may also be associated with RA, including sphingosine 1-phosphate (S1P) receptor [32, 33], A3 adenosine receptor [34, 35] , A2A adenosine receptors [36], purinergic receptor P2X4 [37] and many chemokine receptors [38-41]. Here we find that in CIA mice, CysLT1 signaling pathway, including CysLT1, cPLA2α and 5-LO, are involved in the pathogenesis of RA. Montelukast, CysLT1 antagonists, could effectively alleviate the condition of RA.

Numerous studies have shown that CysLT1 signaling pathway are widely involved in the of respiratory diseases and inflammatory diseases, while their role in autoimmune diseases are rarely explored. Our reports had previously indicated that CysLT1 signaling pathway participated in experimental autoimmune encephalomyelitis (EAE) pathogenesis, blocking CysLT1 signaling pathway with montelukast could effectively ameliorate clinical symptoms of EAE [22].

Then we wondered that whether the CysLTs signaling can also involve in the pathogenesis of RA. Our study indicated that in CIA animals, the synthesizing enzymes, cPLA2α and *5-LO* were up-regulated and *LTC4S* had no significant change. Meanwhile, the expression level of signaling receptor, *CysLT1*, was significant up-regulated. To confirm the relationship of CysLTs signaling with CIA, we checked the ligand levels and found that CysLTs were also higher in CIA than in control mice.

We then detected the effects of CysLT1 antagonist on CIA mice. We found that montelukast, targeted CysLT1, effectively ameliorated clinical symptoms, while CysLT1 signaling did not participate in the proliferation and differentiation of T helper cells, though the trends of decrease could be observed. Plenty of studies indicated that CysLT1 signaling might affect the pathogenesis of disease by inducing chemotaxis of T cells [42-44], our result showed that obvious infiltration of pathogenic T cells in CIA mice can be blocked by CysLT1 antagonists.

Since the Th17 cells and IL-17 have been shown a momentous role in the pathogenesis of CIA, we then tested the cytokine expression level in serum and in lesions joints. The results showed that an increased IL-17 production in serum and hind paw of CIA mice and the gene expression were decreased after treated with CysLT1 antagonist.

Taken together, our findings indicate that the CysLT1 signaling pathway participates in the pathogenesis of CIA and it will alleviate the disease symptoms of CIA after blocking the CysLTs signaling pathways. These results not only reveal the pathogenesis of CIA, but also provide a novel therapeutic target for the treatment of RA.

## MATERIALS AND METHODS

### Animals

Male DBA mice were purchased from Shanghai Laboratory Animal Centre (Shanghai, China). All mice were housed in the Gannan medical University animal care facility and were maintained in pathogen-free conditions. Mice were 7-9 weeks old at the initiation of the experiment and were maintained on standard laboratory chow and water ad libitum. All experiments were approved and conducted in accordance with the guidelines of the Animal Care Committee of Gannan medical University.

### Reagents

Montelukast were from Merck. M-MLV Reverse Transcriptase and RNasin Ribonuclease inhibitor were from Promega (Fitchburg, WI). SYBR Green JumpStart™ Taq ReadyMixTM kit and sodium fluorescein were from Sigma (St.Louis, MO). Fluorescein isothiocyanate (FITC) anti-mouse CD45, FITC anti-mouse CD8a, phycoerythrin (PE) anti-mouse CD45R (B220) and APC anti-mouse/rat Foxp3 staining set were purchased from eBioscience (San Diego, CA). PE-Cy7 anti-mouse CD4, PE anti-mouse IL17A, APC anti-mouse IFN-γ, PE anti-mouse Foxp3 and BD Cytofix/Cytoperm kit were purchased from BD bioscience (Franklin Lakes,NJ). IL-17A, IFN-γ, TGF-β, TNF-α, IL-1β ELISA kit were from Dakewe (Shenzhen, China).

### CIA induction and drug treatment

Chicken type II collagen was dissolved in 0.05M aceticacid at a concentration of 2 mg/ml by stirring overnight at 4°C and was then emulsified in an equal volume of CFA. Collagen (100 μg) was injected intradermally at the base of the tail and boost at day 21. CIA was scored from day 1 post booster using a scale of 0-4 per limb, where 0=no swelling or redness, 1 =swelling or redness in 1 digit, 2 =mild swelling and redness involving the entire paw, 3 =moderate swelling and redness involving the entire paw, and 4 =severe swelling and redness involving the entire paw. For drug treatment, montelukast was injected intraperitoneally (30 mg/kg body weight in saline) once daily from day 1 post booster till the end of the study. Saline was given as a vehicle control (100 μl for each mouse).

### Histopathological and immunofluorescent analysis

For analysis of drug effects, hind paw was collected after PBS perfusion. For histological staining, mice were anesthetized and perfused with PBS (pH 7.4) followed by 4% (w/v) paraformaldehyde. Tissue samples were then fixed in 4% (w/v) paraformaldehyde overnight and then decalcified for 7 days with 15% EDTA-2Na, embedded in paraffin. Section of hind paw were stained with hematoxylin and eosin for analysis of inflammation, respectively. Sections of hind paw were stained with anti-mouse CD4 antibody and anti-mouse IL-17 antibody (BioLegend) and followed with appropriate fluorescent-labeled secondary antibody.

### Reverse transcription and real-time PCR

Total RNA was extracted from mouse tissues using TRIzol (Invitrogen) according to the manufacturer’s instructions. The RNA was subjected to reverse transcription with random hexamer primer and M-MLV Reverse Transcriptase (Progema). Real-time PCR was conducted in the LightCycler quantitative PCR apparatus (Stratagene) using the SYBR Green JumpStart™ Taq ReadyMix™ kit (Sigma). Expression values were normalized to β-Actin. The primer pairs used are as follows: CysLT1 sense- CTCCAAGGCACCAAGCAGAC, CysLT1 anti-sense- TGCCAAAGAAACCCACAACAG; 5-LO sense- CACGCATCTGGTGTCTGAGG, 5-LO anti-sense- CCTTAGTGTTGATAGCAATGGTGA; cPLA2α sense- GACAGCAGGAAGCGAACGAGAC, cPLA2α anti-sense-CGTAGTTGGCATCCATCAGTGTGA, LTC4S sense- CCTGTGCGGACTGTTCTACCT, LTC4S anti-sense- GCCATCGCCACCAGCA; β-actin sense- GGCTGTATTCCCCTCCATCG, β-actin anti-sense- CCAGTTGGTAACAATGCCATGTT; IL-17A sense- TTAACTCCCTTGGCGCAAAA; IL-17A antisense- CTTTCCCTCCGCATTGACAC; TGF-β sense-CTCCCGTGGCTTCTAGTGCT; TGF-β antisense-AGCCTTAGTTTGGACAGGATCTG; IFN-γ sense- ACAATGAACGCTACACACTGCAT; IFN-γ antisense-CCTTTTGCCAGTTCCTCCAG; IL-1βsense- AAGCCTCGTGCTGTCGGA; IL-1βantisense-CAGGGTGGGTGTGCCGT

### Cysteinyl leukotriene EIA

The CysLTs in serum were quantified by using a competitive enzyme immunoassay (EIA, Cayman chemical), according to the manufacturer’s instructions. Orbital blood was collected and incubated at 4°C for 30 min and serum was collected after 10 min centrifugation at 4500g. hind paw and lymph node homogenate supernatant were collected after 10 min centrifugation at 12000g. data were calculated as pg or ng of CysLTs per ml liquid and then normalized to the naïve mice.

### CD4^+^ T cell separation and *in vitro* differentiation

Naive CD4^+^ T cells were prepared by magnetic cell separation (invitrogen) from spleens of male DBA mice 8-9 weeks of age. Separated cells were activated with anti-CD3 (2 µg/ml; 145-2C11, soluble; BD Pharmingen) and anti-CD28 (2 µg/ml; 37.51, soluble; BD Pharmingen) and were induced to differentiate into Th1 cells by supplementation with IL-12 (10 ng/ml; Peprotech) and anti-IL-4; or into inducible Treg cells with TGF-β1 (5 ng/ml; Peprotech), recombinant mouse IL-2 (50 U/ml; Peprotech) and anti-IFN-γ. For Th17 differentiation, cells received anti-IL-4 and anti-IFN-γ plus a Th17 ‘cocktail’ containing TGF-β1 (3 ng/ml), IL-6 (30 ng/ml; eBioscience), tumor necrosis factor (10 ng/ml; Peprotech) and IL-1β (10 ng/ml; Peprotech). Neutralizing anti-IFN-γ (XMG1.2; BD Pharmingen) and anti-IL-4 (11B11; BD Pharmingen) were each used at a concentration of 10 μg/ml. Compounds at various concentrations were added with the cytokine cocktail to assess their influence on T cell differentiation.

### Flow cytometry

Splenocytes or leukocytes in peripheral blood were incubated for 5 hr at 37°C with phorbol 12-myristate 13-acetate (50 ng/ml; Sigma), ionomycin (750 ng/ml; Sigma) and brefeldin A (1.0 μg/ml; Sigma). Surface markers were stained with relevant antibodies. After surface staining, cells were re-suspended in fixation/permeabilization solution (Cytofix/Cytoperm kit; BD Pharmingen) and intracellular cytokine staining was done according to the manufacturer’s protocol. For Foxp3 staining, cells were not stimulated with phorbol 12-myristate 13-acetate and ionomycin; instead, the protocol for the Foxp3 Staining Buffer set was followed the manufacturer’s instructions (eBioscience). Data was acquired using a BD LSRII Flow Cytometer (BD Bioscience) and analyzed using FlowJo software (Tree Star).

### ELISA

Control or CIA mice treated with vehicle or montelukast, respectively were sacrificed at week 9 or 12 post booster immunization. The serum was collected and the hind paw through tissue homogenization. Supernatants were collected. The cytokines (IL-17A, IFN-γ, TNF-α, IL-1β and TGF-β) in serum and in supernatant were determined using specific ELISA Kits (Dakewe, Shenzhen, China), according to the manufacturer’s instructions.

### CFSE staining

T cells were labelled with 5 μm CFSE (Invitrogen, CA, USA) at 37 °C for 10 min. Then, the cells were thoroughly washed with 1% FBS/PBS to remove the excess dye according to the manufacturer instructions. Labeled cells were seeded (5x10^5^) in 6 well plates and cultured for 72 hours in a 37°C humidified atmosphere with 5% CO_2_. Data was acquired using a BD LSRII Flow Cytometer (BD Bioscience) and analyzed using FlowJo software (Tree Star).

### Western blot

Cells were washed once with PBS, lysed in lysis buffer (50 mM Tris [pH 7.4], 150 mm NaCl, 0.1% CHAPS, 1 mm EDTA, 1 mm NaF, 1 mm Na_3_VO4, and protease inhibitors) by sonication for 30s on ice. Protein concentrations were determined by the BCA method (Thermo Scientific). Equal amounts of protein samples were subjected to SDS-PAGE and then transferred onto polyvinylidene difluoride membranes (Millipore). Western blotting was performed using the mouse anti-Bcl-2 , anti-Caspase-3 and corresponding HRP-conjugated secondary Ab (Promega)

### Statistical analysis

Data are presented as mean±SEM. The statistical significance of the CIA clinical scores between treatments was analyzed with two-way ANOVA test. And the CIA scores at a given date were analyzed by Mann-Whitney test. Other analyses, including gene expression, cytokine production, were assessed by Student’s t-test or two-way ANOVA test. *p* < 0.05 was considered statistically significant.

## SUPPLEMENTARY MATERIALS FIGURES




