# Supplementary material for: CysLT1 receptor antagonist alleviates pathogenesis of collagen-induced arthritis mouse model
Source: Oncotarget. 2017 Nov 26;8(65):108418–29. doi: 10.18632/oncotarget.22664 (PMC5752453; doi:10.18632/oncotarget.22664)
Supplement: Supplementary file 1 [file oncotarget-08-108418-s001.pdf]

CysLT1 receptor antagonist alleviates pathogenesis of collagen-induced arthritis mouse model

SUPPLEMENTARY MATERIALS

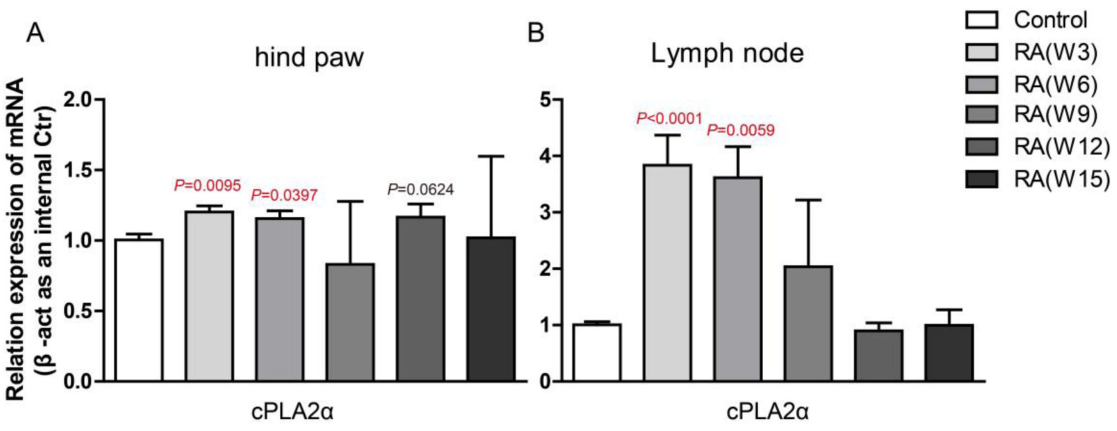

Supplementary Figure 1: Up-regulation of cPLA2α during CIA pathogenesis. mRNA was isolated from hind paw, lymph node of control mice and CIA mice at weeks 3, 6, 9, 12, 15 post booster immunization. qPCR was performed to analyze gene expression. Results were normalized to β-actin expression in the same sample and then normalized to the control. *cPLA2α* gene expression in hind paw (**A**), lymph node (**B**). Data are presented as mean ± SEM (n=6) and are representative of two independent experiments.

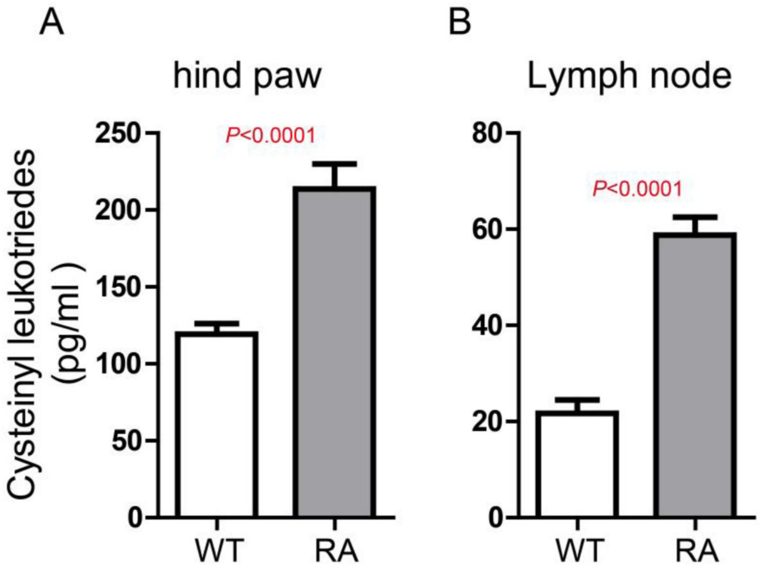

Supplementary Figure 2: Significantly increased CysLTs levels in the hind paw and lymph node of CIA mice. Total CysLTs concentration in hind paw (**A**) and lymph node (**B**) (6 weeks post booster immunization) were measured with an EIA kit. Data are presented as mean ± SEM (n=3) and are representative of three independent experiments.

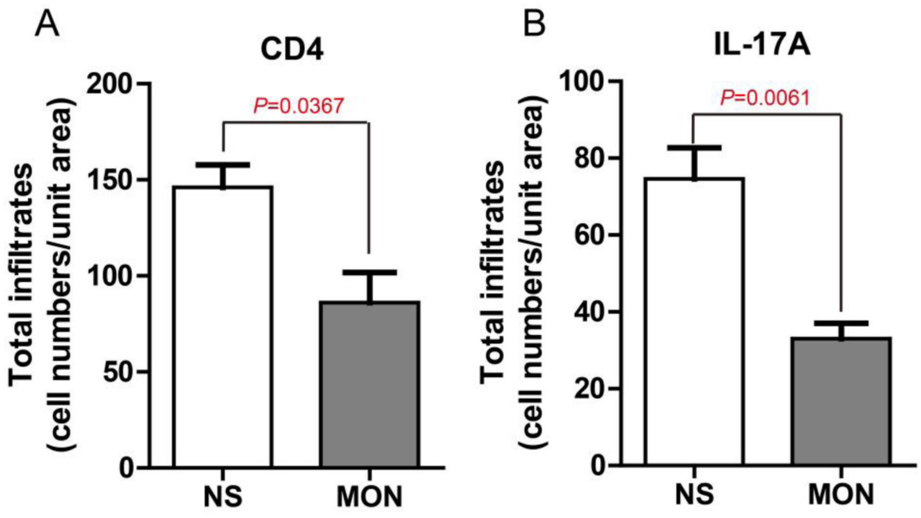

Supplementary Figure 3: Montelukast treatment decreased CD4+ T cells (A) and IL-17A+ T cells (B) infiltrating in unit area in the hind paw sections of CIA mice. Data are presented as mean ± SEM (n=3) and are representative of three independent experiments.

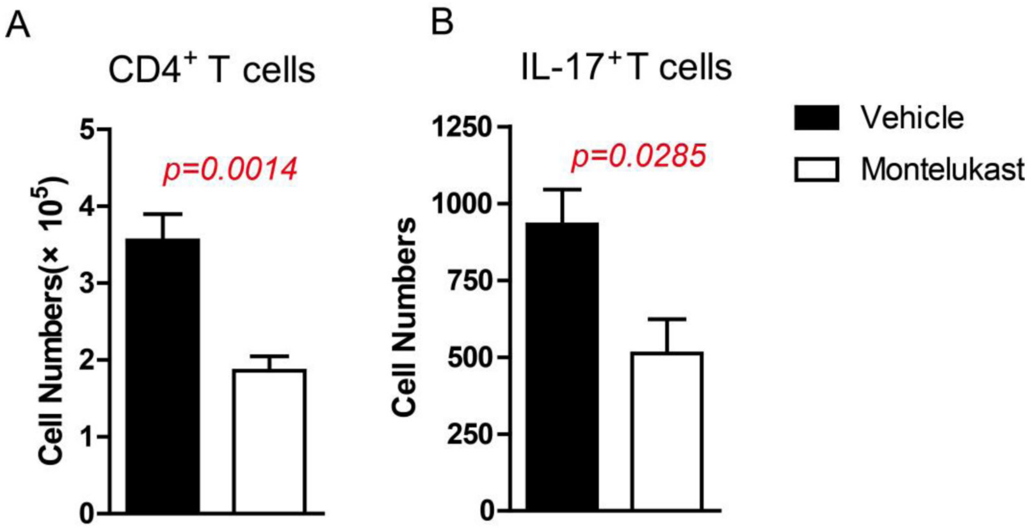

Supplementary Figure 4: Montelukast treatment decreased CD4+ T cells (A) and IL-17A+ T cells (B) infiltrating in synovial fluid in CIA mice. Data are presented as mean ± SEM (n=3) and are representative of three independent experiments.

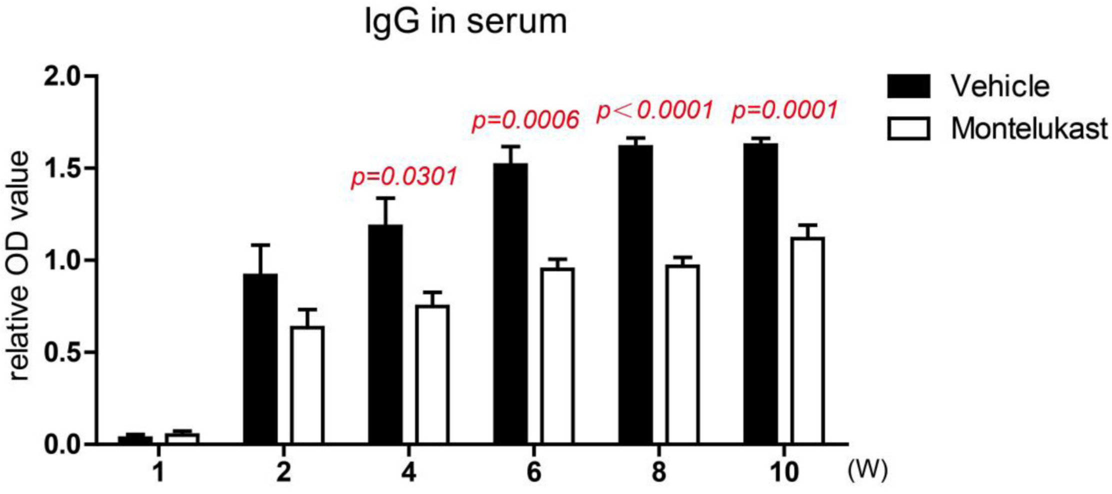

Supplementary Figure 5: Montelukast treatment could decrease the levels of anti collagen II auto-Abs. The levels of anti collagen II auto-Abs were tested by ELISA between 2 experimental groups from serum. Data are presented as mean ± SEM (n=3) and are representative of three independent experiments.

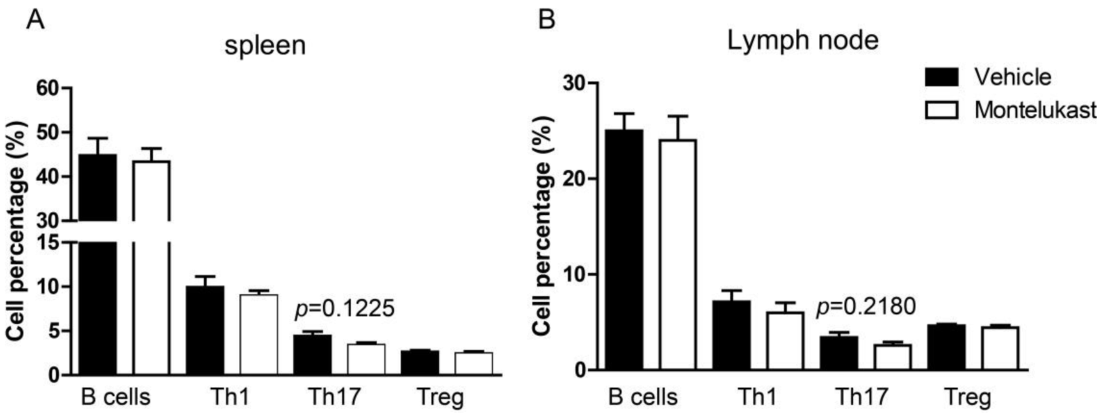

Supplementary Figure 6: Montelukast treatment did not affect T cell proliferation in spleen and lymph node. Surface staining and intracellular staining show that there were no differences between 2 experimental groups in spleen (**A**) and lymph node (**B**) . Data are presented as mean ± SEM (n=3) and are representative of three independent experiments.

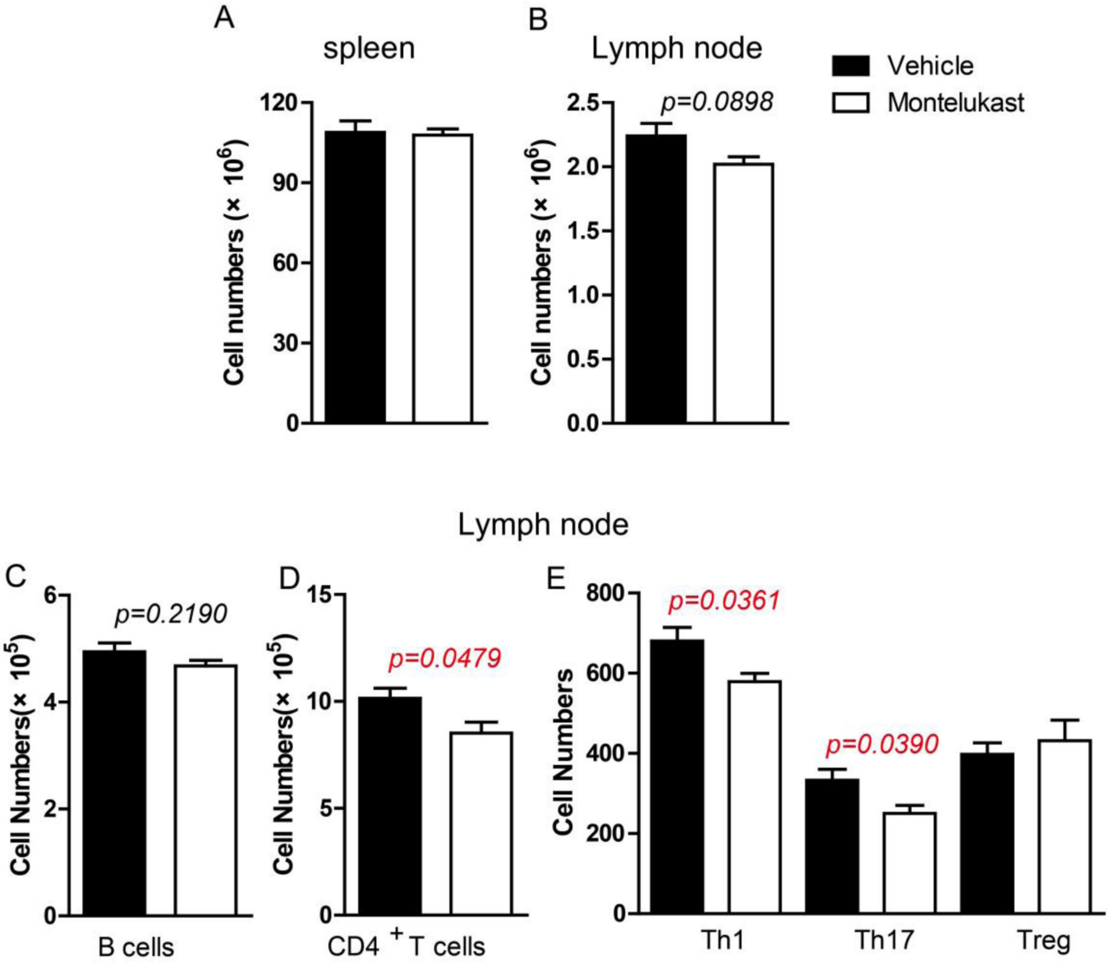

Supplementary Figure 7: Montelukast treatment could reduce T cell indwelling in lymph node. Absolute cell numbers in spleen (**A**) and lymph node (**B**) were checked and the trend of decrease in lymph node was observed. The absolute cell numbers of CD4+ T cells (**D**), Th1 and Th17 (**E**) were decreased in montelukast treated CIA mice. Data are presented as mean ± SEM (n=3) and are representative of three independent experiments.

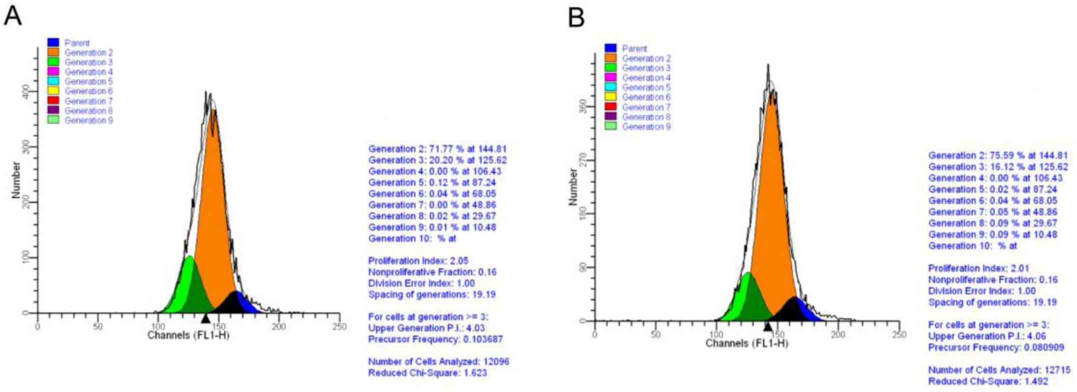

Supplementary Figure 8: Montelukast treatment did not decrease the proliferation of T cell in lymph node. CFSE labeling shows that there were no differences between vehicle treatment group (**A**) and montelukast treatment group (**B**) . Data are presented as mean ± SEM (n=3) and are representative of three independent experiments.

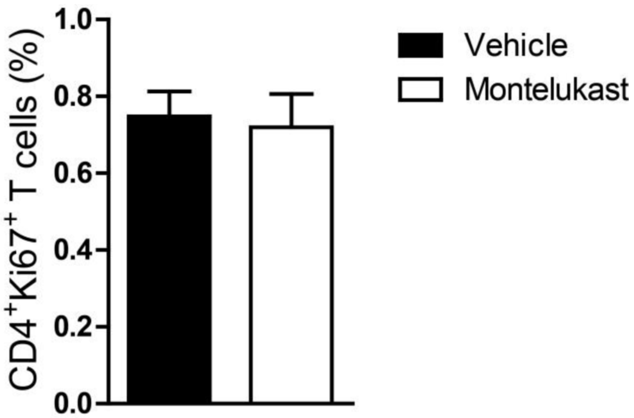

Supplementary Figure 9: Montelukast treatment did not influence cell proliferation in lymph node. $CD4^{+}Ki67^{+}$ T cells were checked by flow cytometry, there were no differences between 2 experimental groups . Data are presented as mean ± SEM (n=3) and are representative of three independent experiments.

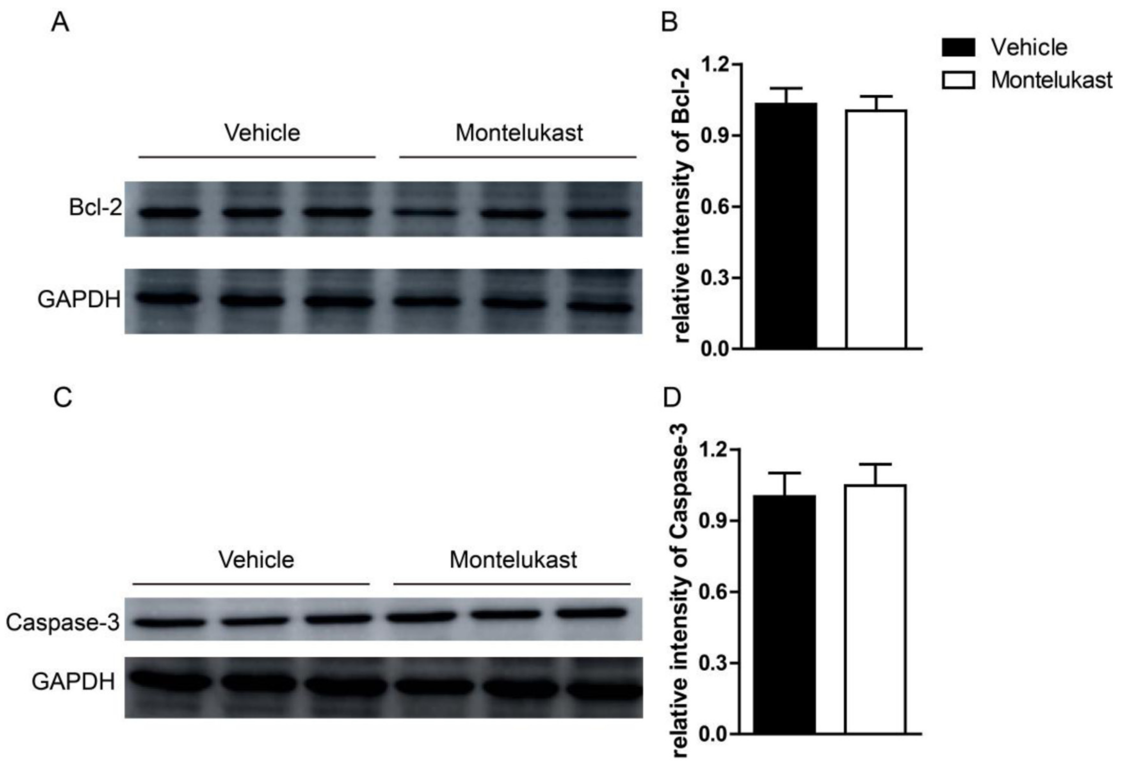

Supplementary Figure 10: Montelukast treatment did not affect cell apoptosis in lymph node. Apoptosis related marker: Bcl-2 (**A**) and Caspase-3 (**B**) we tested by Western blotting, there were no differences between 2 experimental groups . Data are presented as mean ± SEM (n=3) and are representative of three independent experiments.

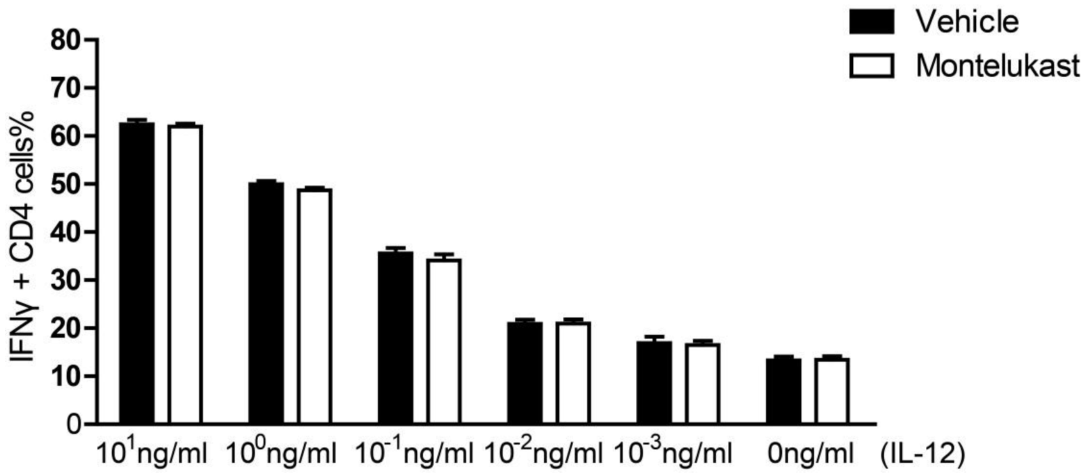

Supplementary Figure 11: IL-12 concentration did not mask the montelukast effect. Different concentration of IL-12 were tried for in vitro Th1 differentiation, there were no differences between 2 experimental groups . Data are presented as mean ± SEM (n=3) and are representative of three independent experiments.

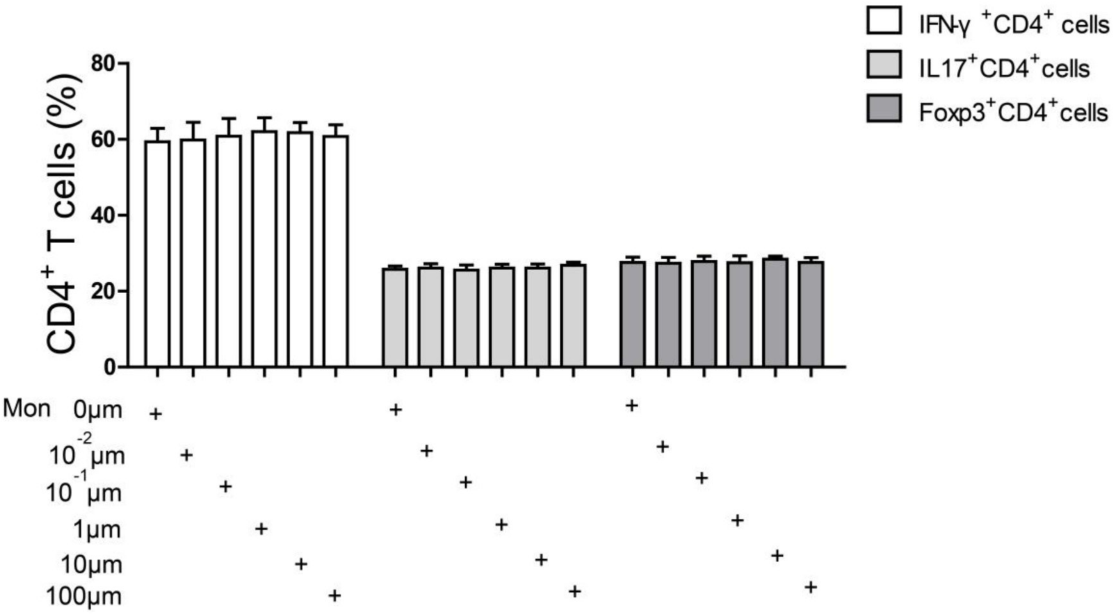

Supplementary Figure 12: Montelukast concentration did not influence the CD4+ T cells differentiation. Different concentration of montelukast were tried for in vitro CD4+ T cells differentiation, there were no differences effect in different concentration of montelukast. Data are presented as mean ± SEM (n=3) and are representative of three independent experiments.

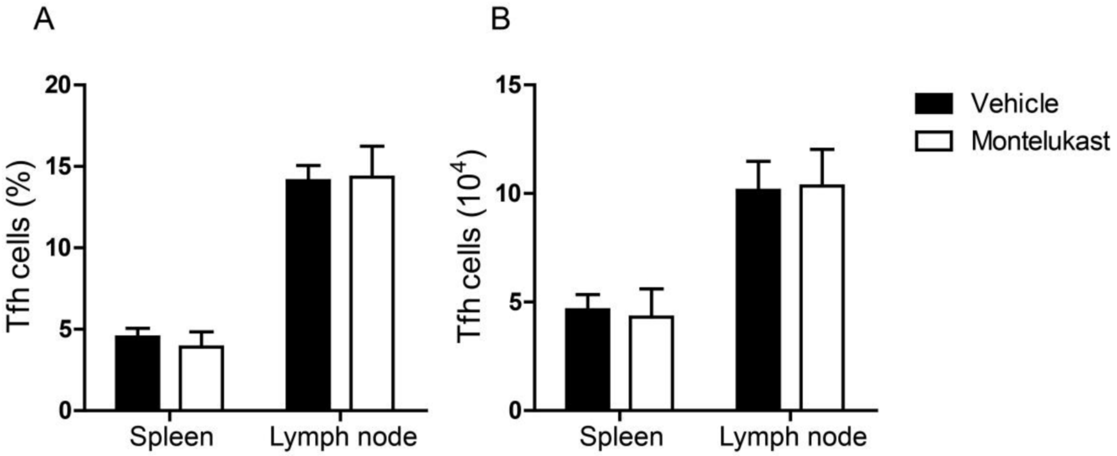

Supplementary Figure 13: Montelukast treatment did not affect Tfh cell percentage and absolute cell numbers in spleen and lymph node. Flow cytometry shows that there were no differences between 2 experimental groups in percentage (**A**) and absolute cell numbers (**B**) in spleen and lymph node . Data are presented as mean ± SEM (n=3) and are representative of three independent experiments.

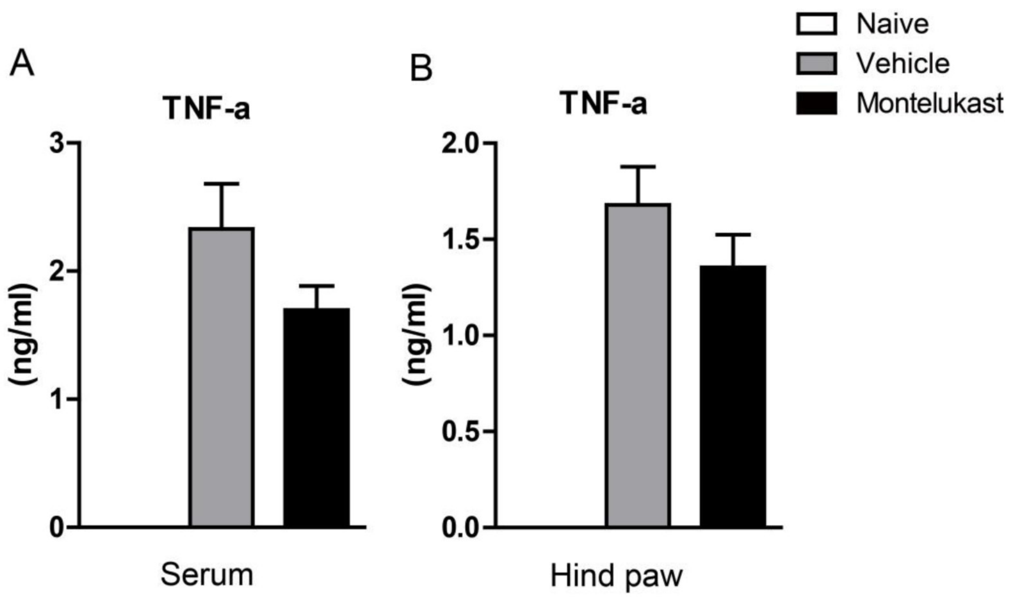

Supplementary Figure 14: Montelukast treatment did not decrease the production of TNF-α. ELISA shows that there were no differences between 2 experimental groups in the production of TNF-α in serum (**A**) and hind paw (**B**). Data are presented as mean ± SEM (n=3) and are representative of three independent experiments.

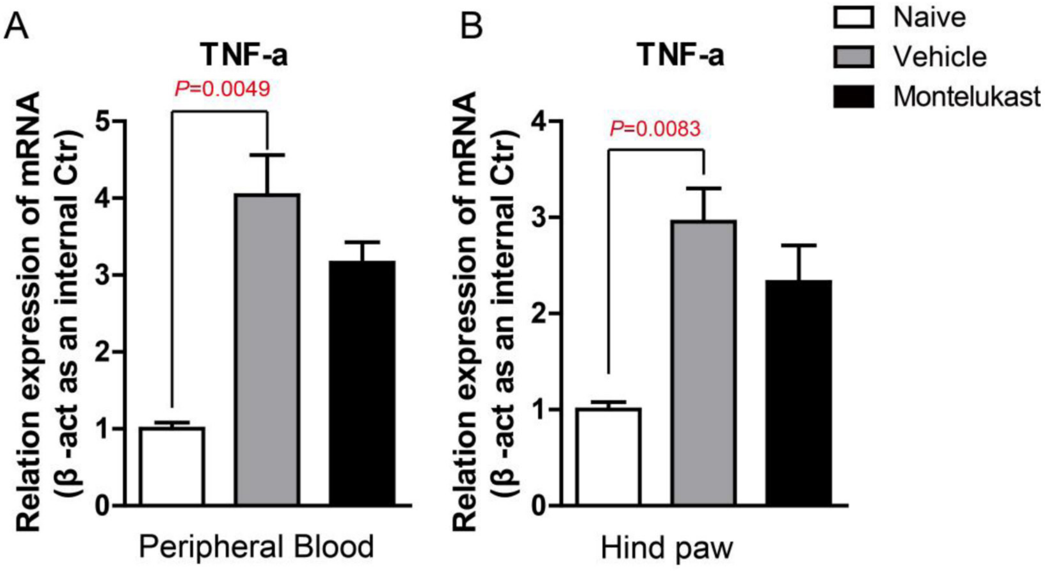

Supplementary Figure 15: Montelukast treatment did not decrease the expression of TNF-α gene. qPCR shows that there were no differences between 2 experimental groups in the expression of TNF-α mRNA in peripheral blood (**A**) and hind paw (**B**). Data are presented as mean ± SEM (n=3) and are representative of three independent experiments.
